# Hepatitis C Virus (HCV)–Apolipoprotein Interactions and Immune Evasion and Their Impact on HCV Vaccine Design

**DOI:** 10.3389/fimmu.2018.01436

**Published:** 2018-06-21

**Authors:** Florian Wrensch, Emilie Crouchet, Gaetan Ligat, Mirjam B. Zeisel, Zhen-Yong Keck, Steven K. H. Foung, Catherine Schuster, Thomas F. Baumert

**Affiliations:** ^1^INSERM, U1110, Institut de Recherche sur les Maladies Virales et Hépatiques, Strasbourg, France; ^2^Université de Strasbourg, Strasbourg, France; ^3^INSERM U1052, CNRS UMR 5286, Cancer Research Center of Lyon (CRCL), Université de Lyon (UCBL), Lyon, France; ^4^Department of Pathology, Stanford University School of Medicine, Stanford, CA, United States; ^5^Institut Hospitalo-Universitaire, Pôle Hépato-digestif, Hôpitaux Universitaires de Strasbourg, Strasbourg, France; ^6^Institut Universitaire de France, Paris, France

**Keywords:** hepatitis C virus, apolipoproteins, neutralizing antibodies, lipo-viro-particle, viral evasion, ApoE

## Abstract

With more than 71 million people chronically infected, hepatitis C virus (HCV) is one of the leading causes of liver disease and hepatocellular carcinoma. While efficient antiviral therapies have entered clinical standard of care, the development of a protective vaccine is still elusive. Recent studies have shown that the HCV life cycle is closely linked to lipid metabolism. HCV virions associate with hepatocyte-derived lipoproteins to form infectious hybrid particles that have been termed lipo-viro-particles. The close association with lipoproteins is not only critical for virus entry and assembly but also plays an important role during viral pathogenesis and for viral evasion from neutralizing antibodies. In this review, we summarize recent findings on the functional role of apolipoproteins for HCV entry and assembly. Furthermore, we highlight the impact of HCV–apolipoprotein interactions for evasion from neutralizing antibodies and discuss the consequences for antiviral therapy and vaccine design. Understanding these interactions offers novel strategies for the development of an urgently needed protective vaccine.

## Introduction

With more than 71 million people chronically infected (1, 2), hepatitis C virus (HCV) is one of the leading causes of liver disease and hepatocellular carcinoma (3). The recent development of direct acting antivirals with sustained virological response rates of over 90% has revolutionized HCV therapy. However, several limitations remain: high treatment costs, emergence of resistant variants, difficult-to-treat patients with significantly decreased sustained virological response rates, and the possibility of reinfection highlight the urgent need for a protective HCV vaccine (4).

Despite the combined efforts of the HCV research community, HCV vaccine design has been hampered by the ability of HCV to rapidly mutate and escape from protective immune responses (5). This is partly due to the intimate relationship of HCV with the host lipid metabolism. All steps of the HCV life cycle are dependent on the interaction with lipoproteins and apolipoproteins. Moreover, the interaction of HCV with lipoproteins leads to the formation of lipo-viro-particles (LVPs), which is critical for HCV infectivity and evasion from neutralizing antibodies. Thus, understanding the role of these interactions is crucial for future vaccine research. Here, we review recent findings on HCV–apolipoprotein interactions, highlight their role for viral escape, and discuss their implications for HCV antiviral therapies and vaccine design.

## The Functional Role of Apolipoproteins in the HCV Life Cycle

### Structure of the LVP, the Infectious HCV Particle

**Figure 1:**
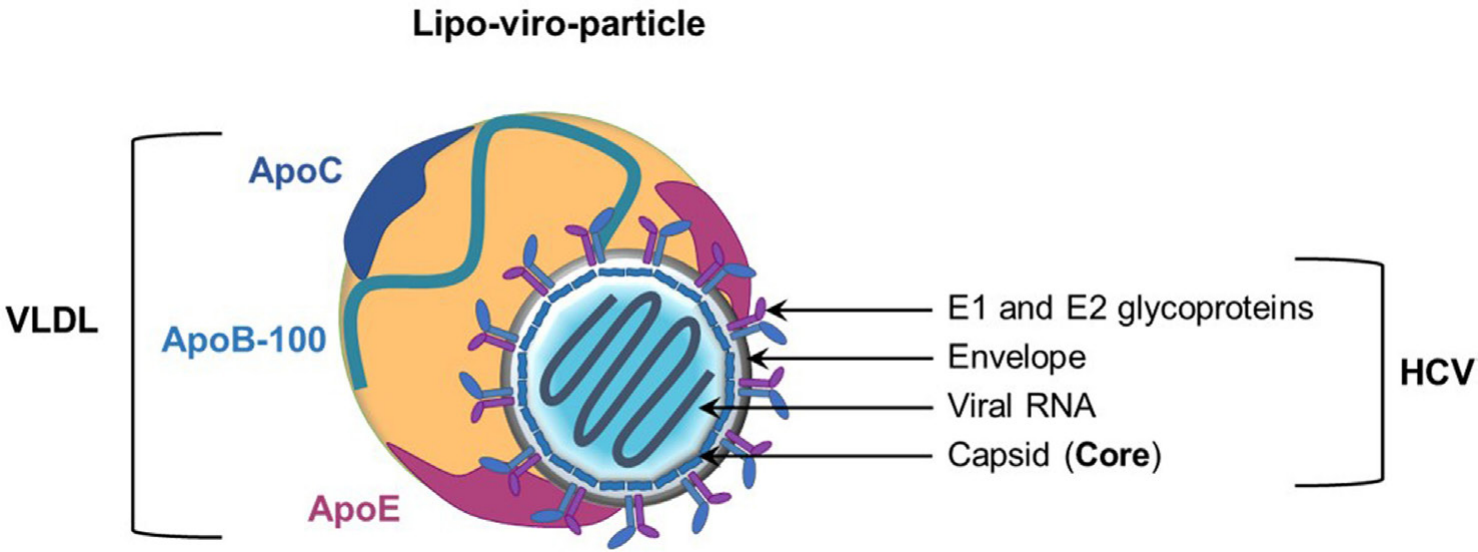
Model of the hepatitis C virus (HCV) lipo-viro-particle (LVP). The HCV particle consists of an icosahedral capsid, formed by the viral core protein, containing the positive-stranded viral RNA. The nucleocapsid is surrounded by an endoplasmic reticulum-derived envelop in which E1 and E2 glycoproteins are embedded. The highly infectious HCV particle corresponds to a hybrid particle composed of very-low-density lipoprotein (VLDL) components and viral components named LVP. The different apolipoproteins classically associated with VLDL and LVP are illustrated on this picture (ApoB-100 and the exchangeable apolipoproteins ApoE and ApoCs).

Hepatitis C virus is an enveloped positive-stranded RNA virus belonging to the *Flaviviridae* family. The viral particle consists of a nucleocapsid containing the viral RNA surrounded by an endoplasmic reticulum (ER)-derived envelope in which viral E1 and E2 glycoproteins are embedded as heterodimers (6) (Figure 1). Over the past years, several studies strongly demonstrated the tight link between HCV and lipid metabolism (7, 8). A hallmark of the virus is its association with host lipoproteins. Indeed, highly infectious HCV particles circulate in patient serum in association with very-low-density lipoproteins (VLDL) or low-density lipoproteins (LDL), to form LVPs (9–11). Consequently, LVPs share several biophysical properties with the VLDL. Infectious LVPs have a low density (between 1.03 and 1.10 g/ml), are rich in cholesterol and triglycerides, and contain apolipoproteins (Apo) such as ApoB, ApoA-I, ApoE, and ApoCs (12–15) (Figure 1). Characterization of HCV particles produced in cell culture (HCVcc) has confirmed these properties (16–18). Interactions of HCV particles with lipoprotein components play a critical role in the viral life cycle and contribute to viral persistence and development of chronic liver diseases (19).

**Figure 2:**
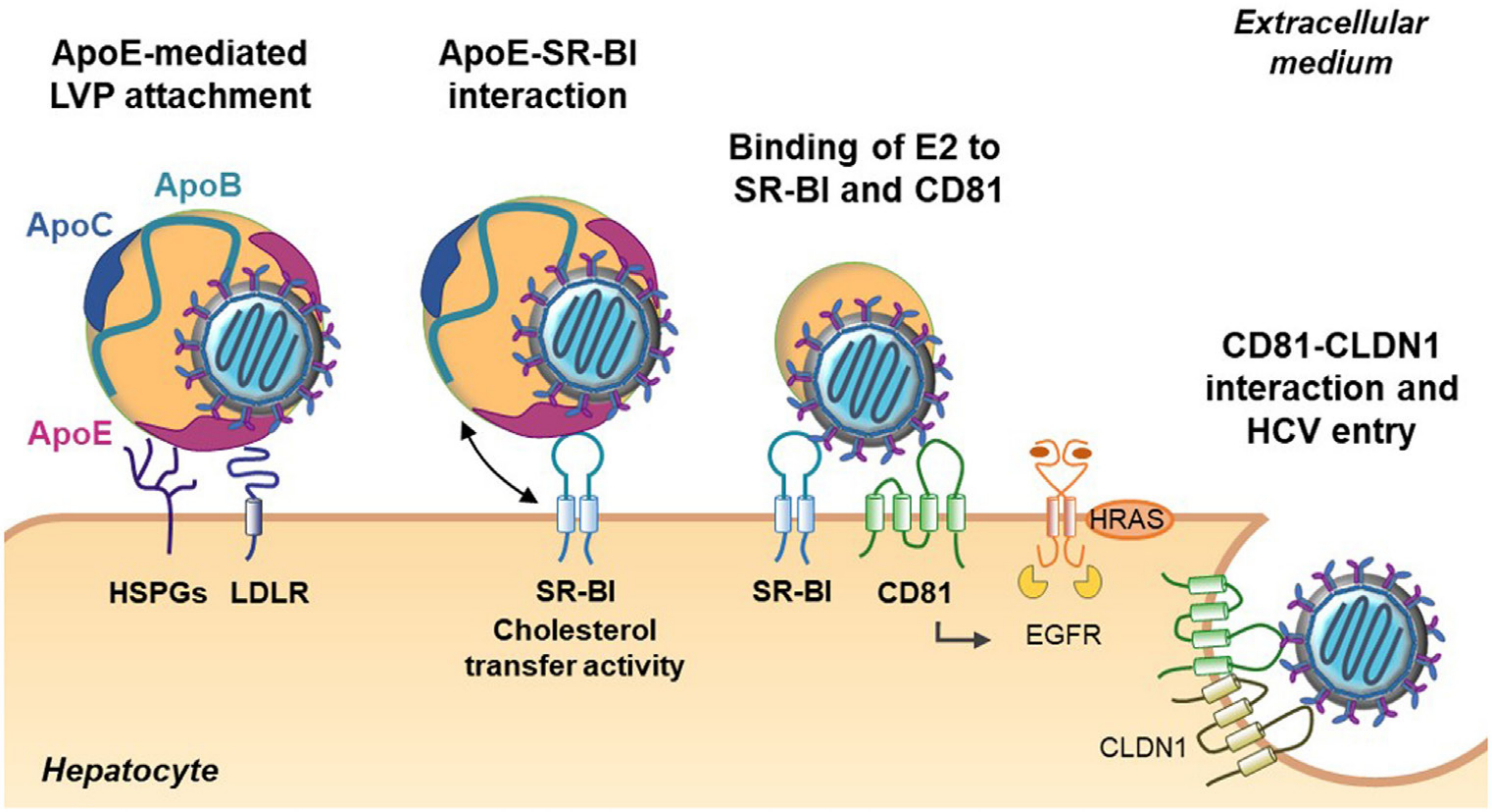
Role of apolipoproteins during early steps of hepatitis C virus (HCV) entry. The first step of HCV entry consists of the interaction between lipo-viro-particle (LVP)-associated ApoE, the heparan sulfate proteoglycans (HSPGs), and the low-density lipoprotein receptor (LDLR). Subsequently, the LVP interacts with the scavenger receptor class B type I (SR-BI) through ApoE and ApoB (not illustrated). The cholesterol transfer activity of SR-BI allows E2 exposure and binding of E2 to SR-BI and the tetraspanin CD81. Binding on CD81 activates the epithelial growth factor receptor (EGFR) signaling pathway and interaction between CD81 and claudin 1 (CLDN1) that triggers HCV entry.

**Figure 3:**
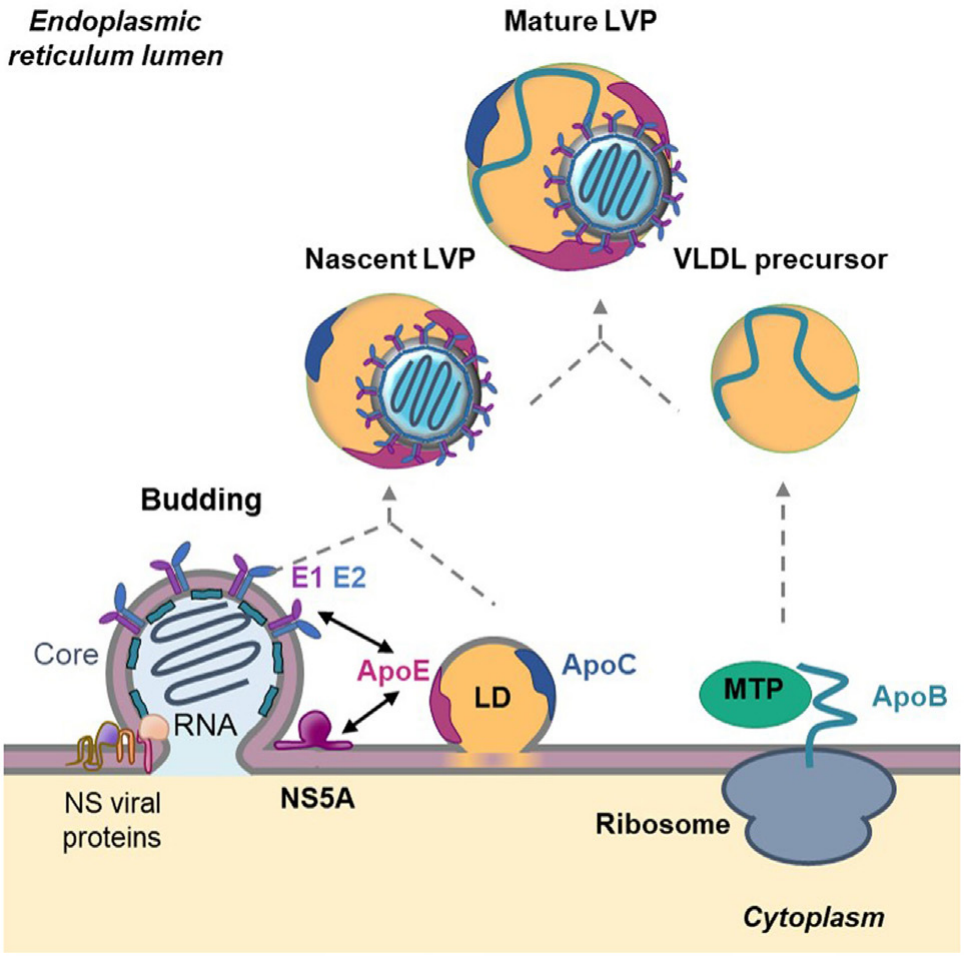
Role of apolipoproteins in hepatitis C virus (HCV) morphogenesis. HCV assembly takes place at the surface of endoplasmic reticulum (ER)-derived membranes in close proximity to lipid droplets (LD). Core protein associates with viral RNA to form the nucleocapsid. The nucleocapsid buds at the ER membrane where E1 and E2 glycoproteins are anchored and afterward associate with nascent LD to acquire ApoE and ApoC. This step is facilitated by the interaction between ApoE and the non-structural (NS) viral protein NS5A as well as by the interaction between ApoE and the glycoproteins E1 and E2. In parallel, ApoB is lipidated by the microsomal triglyceride transfer protein (MTP) to generate very-low density lipoprotein (VLDL) precursors. The nascent HCV particle associates with these precursors by an unknown mechanism to generate mature lipo-viro-particles (LVPs).

Apolipoproteins represent the protein moiety of the lipoproteins. Physiologically, they have three major functions in the lipoprotein metabolism: (i) they stabilize the lipoprotein structure and solubilize the lipid fraction, (ii) they interact with lipoprotein receptors and participate in lipoprotein clearance, and (iii) they act as cofactors for specific enzymes involved in lipoprotein metabolism (20, 21) (Table 1). In many aspects, HCV takes advantage of host apolipoproteins for efficient propagation in hepatocytes (22). The role of apolipoproteins in the HCV life cycle is highlighted in Table 1 and Figures 2 and 3.

**Table 1:** Role of the major apolipoproteins in the HCV life cycle.

Name	Physiological role	Role in HCV life cycle	Reference
*ApoA-I* (exchangeable apolipoprotein)	*Structural role*: major component of HDL	*Structural role*: component of the LVP	(12, 15, 19, 49, 50)
	*HDL metabolism*: involved in HDL maturation by activating LCAT	*HCV morphogenesis*: redundantly participate in the production of infectious HCV particles	
	*Reverse cholesterol transport*: from peripheral tissues to liver through interaction with SR-BI and ABCA1 (cholesterol efflux)		

*ApoB-100* (non exchangeable apolipoprotein)	*Structural role*: major component of VLDL and LDL	*Structural role*: major component of the LVP	(12, 15, 19, 33, 34, 41, 42, 49)
	*Triglyceride transport*: involved in VLDL synthesis and clearance through interaction with LDLR	*HCV entry*: mediates LVP binding through interaction with SR-BI	
	*Cholesterol transport*: transfer of LDL-cholesterol in cells through LDLR	*HCV morphogenesis*: LVP synthesis and secretion	

*ApoC-I* (exchangeable apolipoprotein)	*Structural role*: component of VLDL and HDL	*Structural role*: component of the LVP	(12, 15, 19, 35)
	*HDL metabolism*: LCAT activator	*HCV entry*: enhance HCV infectivity through complex interaction with SR-BI	

*ApoC-II* (exchangeable apolipoprotein)	*Structural role*: component of VLDL and HDL	*Structural role*: component of the LVP	(12, 15, 19, 37)
	*Triglyceride metabolism*: LPL activator	*HCV entry*: physiological HCV entry inhibitor by activating LPL	
		*HCV morphogenesis*: redundantly participate in the production of infectious HCV particles	

*ApoC-III* (exchangeable polipoprotein)	*Structural role*: component of VLDL and HDL	*Structural role*: component of the LVP	(12, 15, 19, 38)
	*Triglyceride metabolism*: LPL inhibitor	*HCV entry*: enhance HCV entry by inhibiting LPL	
		*HCV morphogenesis*: redundantly participate in the production of infectious HCV particles	

*ApoE* (exchangeable apolipoprotein)	*Structural role*: major component of VLDL and HDL	*Structural role*: major component of the LVP	(12, 15, 19, 23–34, 43–61)
	*Triglyceride transport*: involved in VLDL synthesis and clearance trough interaction with HSPG, LRP1, and LDLR	*HCV entry*: mediates LVP binding through interaction with HSPG, LDLR, and SR-BI. Involved in cell-to-cell transmission	
	*HDL metabolism*: involved in reverse cholesterol transport	*HCV morphogenesis*: crucial role in HCV assembly by interaction with NS5A, E1, and E2, necessary for the production and maturation of infectious HCV particles	

*ABCA1, ATP-binding cassette A1; Apo, apolipoprotein; HDL, high-density lipoprotein; HSPG, heparan sulfate proteoglycan; LCAT, lecithin cholesterol acyltransferase; LDL, low-density lipoprotein; LDLR, low-density lipoprotein receptor; LPL, lipoprotein lipase; LRP1, LDLR-related protein 1; LVP, lipo-viro-particle; SR-BI, scavenger receptor class B type I; VLDL, very-low-density lipoprotein; HCV, hepatitis C virus*.

### Role of Apolipoproteins in HCV Entry

The initiation of HCV entry is triggered by LVP binding to cell surface heparan sulfate proteoglycans (HSPGs). Interestingly, it was demonstrated that ApoE plays a role in this process by interacting with syndecan 1 and syndecan 4 HSPGs (23–27). Other groups have highlighted the key role of ApoE in HCV entry. Indeed, HCV infectivity can be efficiently blocked by anti-ApoE antibodies or human synthetic peptides derived from the ApoE receptor-binding domain (18, 25, 28, 29). Moreover, Owen and collaborators observed that ApoE facilitates HCV entry by interacting with the LDL receptor (30). The scavenger receptor class B type I (SR-BI) is another lipoprotein receptor identified as a HCV receptor involved in different steps of HCV entry (31, 32). During the early steps, LVP attachment to SR-BI is mediated by ApoB-100 and ApoE (33, 34). This interaction is thought to induce lipoprotein–HCV dissociation and to expose the viral glycoprotein E2 for direct interaction with SR-BI and the tetraspanin CD81 (32). Of note, it was reported that high-density lipoprotein (HDL)-associated ApoC-I, a natural ligand of SR-BI, improves this step by increasing the fusion rates between viral and target membranes through direct interaction with the LVP (35).

Lipo-viro-particle entry into hepatocytes is also influenced by changes in lipoprotein composition. One of the best examples is the action of the lipoprotein lipase (LPL) on lipoprotein-associated triglycerides. By hydrolyzing triglycerides, LPL decreases the size of the particles and induces a loss of LVP-associated ApoE. The loss of ApoE results in a strong decrease in LVP infectivity (36, 37). Consistent with this finding, it was demonstrated that ApoC-II, the natural activator of the LPL, is an anti-HCV factor, whereas ApoC-III, an LPL inhibitor, facilitates chronic HCV infection (38, 39) (Figure 2).

### Role of Apolipoproteins in HCV Morphogenesis and Maturation

Following HCV entry and viral RNA replication, virions are assembled in a coordinated and complex process (39) (Figure 3). As mentioned above, LVP share numerous properties with VLDL suggesting that HCV coopts the VLDL machinery for its own morphogenesis. In hepatocytes, the VLDL production is divided in two steps. First, ApoB-100 is lipidated by the microsomal triglyceride transfer protein (MTP) to form a VLDL precursor. Second, the precursor is enriched in lipids and acquires ApoE and ApoCs in the ER by a mechanism not fully understood (40). Interestingly, it was shown that the impairment of VLDL synthesis, through MTP inhibitors or siRNA-mediated knockdown of ApoB expression, leads to a decrease in HCV production (12, 41, 42). However, the functional importance of ApoB in HCV assembly remains controversial. Other studies revealed that HCV assembly is independent on ApoB expression but is rather highly dependent on ApoE (43, 44). The different observations could be due to the use of different models and to a defect of Huh7 cells in producing authentic VLDL (45). The hypothesis is supported by studies showing that there is no correlation between the ability to generate VLDL and the production of infectious viral particles. Indeed, the ectopic expression of ApoE but not ApoB is necessary to produce infectious HCV particles in human non-liver cells (46, 47). Recently, two studies demonstrated that ApoE but not ApoB is required for HCV cell-to-cell transmission (47, 48). Finally, by using Huh7 cells knockout for either or both *APOB* and *APOE*, the Matsuura group revealed that ApoB and ApoE redundantly participate in the formation of infectious HCV particles (49). Of note, not only the expression of ApoE but also of other exchangeable apolipoproteins belonging to the ApoA and ApoC family rescued the production of infectious virions, indicating that exchangeable apolipoproteins possess redundant roles in HCV assembly (49, 50). ApoA, ApoC, and ApoE are exchangeable apolipoproteins that are able to dissociate from one lipoprotein and reassociate with another due their high content in α-helical structures (20). Accordingly, two research groups highlighted the role of α-helical structures in HCV morphogenesis. The authors demonstrated that expression of short sequences containing amphipathic α-helices derived from apolipoproteins but also of other proteins such as the human cathelicidin antimicrobial peptide is sufficient to rescue the production of infectious HCV particles in apolipoprotein knockout cells (49–51). Of note, a recently published paper showed that α-helices found in host-derived apolipoproteins and in NS1 of other *Flaviviridae* may have overlapping roles in the formation of infectious flaviviral particles (52).

Despite the redundant role of exchangeable apolipoproteins in HCV morphogenesis, ApoE remains critical for HCV assembly and infectivity. The role of ApoE was reinforced by a study showing that all the main HCV genotypes (from genotypes 1 to 7) are strictly ApoE dependent, regardless of ApoE isoforms. Indeed, the three main ApoE isoforms, ApoE3, ApoE2, and ApoE4, differing at only two amino acid positions (residues 112 and/or 158) seem to complement HCV production to a comparable degree.

The molecular mechanism by which ApoE associates with HCV particles was recently highlighted. Indeed, ApoE was found to interact with the viral protein NS5A through its C-terminal α-helix domain (53, 54). Furthermore, two other studies evidenced the interaction between ApoE and the HCV glycoproteins E1 and E2 in the ER but also at the LVP surface. Association of ApoE with the viral proteins NS5A, E1 and E2 would trigger LVP morphogenesis (55, 56). Finally, it was shown that extracellular ApoE play a role in LVP maturation. Mature LVP are highly enriched in ApoE compared with normal VLDL (18, 57). Recent studies related that ApoE exchange occurs between LVP and circulating lipoproteins. This process is important to maintain a high ApoE level on the LVP surface that is required for an efficient infectivity and facilitates escape host immunity (57–59). Indeed, a study performed in our lab demonstrated that association of ApoE with E2 helps the virus to escape from patient neutralizing antibodies (60). These observations are of utmost importance for vaccine development: design of immunogens mimicking the E2/ApoE interface might help to achieve an efficient neutralizing humoral immune response against HCV (60).

## Apolipoproteins and Viral Pathogenesis

Clinical evidence indicates that chronic HCV infection is associated with dysregulated circulating lipoproteins and apolipoproteins within the HCV-infected hepatocytes. HCV infection induces the accumulation of lipoproteins and apolipoproteins by upregulation of genes involved in lipid synthesis (61, 62). Disturbed lipoprotein and Apo homeostasis may not only contribute to clinical progression of HCV-induced liver diseases but also represent important risk factors for cardiovascular disease (63).

Hepatitis C virus infection appears to disturb serum apolipoprotein levels depending on the genotype of the virus. Seki et al. reported that infection with genotype 1b was associated with elevated serum levels of ApoA-II and ApoE and reduced levels of ApoC-II and ApoC-III. By contrast, genotype 2 infection only reduced ApoC-II and ApoC-III serum levels. In infected patients, a reduction in ApoC-II and ApoC-III serum levels may enhance HCV infection. In particular, low ApoC-II serum levels were found to be associated with advanced liver fibrosis, which indicate an important role in liver pathogenesis (64). A similar effect has been observed for the HCV core protein induced upregulation of ApoC-IV that has been also reported to induce hepatic steatosis (65).

Finally, it has been observed that single-nucleotide polymorphisms (SNPs) in apolipoproteins are associated with HCV infection and alteration of lipid metabolism. Two studies reported that SNP rs12979860 near the IL28B gene, which encodes for interferon-λ-3, is associated with the response to IFN treatment (66, 67). A follow-up study reported that the rs12979860 CC responder genotype was associated with higher serum levels of ApoB, suggesting that alteration of ApoB levels are part of the IFN response (68). Moreover, a recent study showed that the ApoB polymorphism rs1042034 is significantly associated with the HCV infection status (69). The AA allele, which was characterized by significantly lower serum levels of LDL-cholesterol, might contribute to facilitating serum LDL uptake into human hepatocytes. Consequently, individuals carrying the polymorphism might be more susceptible to HCV infection, indicating a direct influence of the polymorphism on the low-density lipoprotein receptor-mediated host cell entry of HCV.

## Mechanisms of Neutralizing Antibodies Targeting HCV Infection

Neutralizing antibodies to HCV are mainly directed at the E2 glycoprotein with a wide range of specificity and degree of conservation. The majority of broadly neutralizing antibodies mediate neutralization by blocking virus binding to CD81, a tetraspanin HCV co-receptor (70). Fine epitope mapping shows that these antibodies are directed at clusters of overlapping epitopes that include key residues that also participate in virus interaction with CD81 (71, 72). Thus, the binding of these antibodies to the viral surface prevents virus interaction to this required co-receptor during viral entry. These antibody clusters are designated as antigenic domains B, D, and E or antigenic region 3 (AR3) (see Keck et al. in this issue). Note that domains B, D, and AR3 are clusters of overlapping conformational epitopes, while domain E has mainly overlapping linear epitopes. Two other clusters of broadly neutralizing antibodies are directed at conformational epitopes formed by key residues on both E1 and E2 glycoproteins, designated as AR4 and AR5. These antibodies do not block virus binding to CD81 and are thought to mediate neutralization by inhibiting E1E2 heterodimer conformational change during the entry process (73).

The N-terminal region of E2 (amino acid 384–410) is hypervariable and some antibodies to this region, designated as HVR1, do mediate virus neutralization. These antibodies are directed at epitopes located at the C-terminal portion of HVR1 that includes key residues that are also found to be involved in the initial attachment step of virus entry to heparan sulfate and subsequent interaction with SR-BI (74–76). While the majority of antibodies to HVR1 are isolate specific, several described antibodies exhibit broad virus neutralization and block virus binding to SR-BI (77, 78).

## Apolipoproteins and Viral Evasion During Virus Neutralization

With about 75–80% of all HCV infections progressing to chronic disease, it is clear that evasion from the neutralizing antibody response is a key feature of HCV: although patients during the chronic phase often have high levels of serum neutralizing antibodies, in most cases, the immune system is not able to control the infection. A potential determinant for viral escape is the close association of HCV with lipid metabolism (79).

Early indications that HCV-lipoprotein interactions are involved in viral escape from the neutralizing antibody response stem from reinfection experiments in chimpanzees, where infection could only be transmitted by low-density fractions of serum-derived HCV (80).

A very similar effect was observed in virus derived from humanized mice: those particles displayed lower density and higher infectivity than cell culture-derived viruses. However, this effect was lost after a single passage in cell culture, indicating the responsibility of host-derived factors (17). Indeed, highly infectious particles were associated with ApoB and E, forming LVPs with a buoyant density of 1.06 mg/ml while poorly infectious LVPs of buoyant densities around 1.25 mg/ml were linked to immunoglobulins (81). Furthermore, Thomssen et al. reported that virus-bound ApoB-100 excluded binding of neutralizing antibodies (82), indicating a negative correlation of apolipoprotein content and binding of neutralizing antibodies.

The development of cell culture-derived HCV (HCVcc) that displays a similar lipid composition as native serum-derived HCV particles allowed for a more detailed analysis of the involved mechanism. Immature intracellular HCVcc that are characterized by a lower lipoprotein content, when compared with released HCVcc were more sensitive to neutralization by anti-E2 antibodies and less sensitive to anti-ApoE antibodies than mature HCVcc (83), indicating a shielding function of the lipoproteins. In addition, a cell culture adaptive mutation in E2 (I414T) that decreased the dependency on the host factors SR-BI and CD81 also led to reduced lipoprotein content in combination with increased susceptibility to neutralizing anti-E2 antibodies (83). A similar mechanism was observed for mutation G451R that also decreased the dependency of HCV on SR-B1 and CD81 and altered the relationship of infectivity and density with peak infectivity occurring at higher density, meaning lower lipid content, than the wild-type virus (84). This was associated with a drastic increase in the sensitivity of the virus to neutralizing antibodies targeting E2 or soluble CD81 protein, indicating that lipoprotein content directly affects the binding efficiency of neutralizing antibodies (84). Bankwitz et al. recently confirmed that physiological levels of ApoE, which are much higher than those found in cell culture (10–60 µg/ml compared with 0.3 µg/ml) directly enhanced HCV particle infectivity across all genotypes (59). Furthermore, the overall ApoE capacity of serum-derived HCV particles was higher than cell culture-derived HCV, indicating that not only the higher concentration of the serum but also apolipoprotein incorporation during the assembly process is responsible for the elevated apolipoprotein levels of native HCV particles. Enhancement of infection was independent of HVR1 and SR-BI but was reliant on HSPGs. Removal of HSPGs abrogated the enhancement of infection by ApoE, indicating that incorporated ApoE mediated the binding to cell surface proteoglycans (59).

A recent publication showed that ApoE levels in HCV-producing cells directly determined the ability of HCV to evade the neutralizing antibody response (60). Viruses that were produced in hepatoma cells expressing only low amounts of ApoE were more susceptible to neutralizing antibodies directed against the envelope proteins. Utilization of ApoE to escape from neutralizing antibodies was pan-genotypic; however, it was most exploited by variants that were characterized by most efficient viral escape (60). Functional studies with different monoclonal antibodies revealed that E2 domains B and C were exposed after ApoE deletion, confirming the shielding mechanism of ApoE. In variants that were selected post liver transplantation, a mutation on E2 residue 447 appeared to modify the E2–ApoE interaction that altered the sensitivity to neutralization by both ApoE and E2-specific neutralizing antibodies, despite comparable incorporation of ApoE in wild-type and mutant viruses (60), indicating that viral evasion mediated by ApoE is determined both by incorporation and conformation of incorporated ApoE. A study by Weller et al. demonstrated that usage of ApoE was strain dependent, indicating that ApoE might contribute to strain-dependent differences in neutralization (85). Shielding of antigenic sites on the envelope proteins, however, is not the only mechanism by which apolipoproteins contribute to viral escape.

It has been shown that lipoproteins attenuated antibody binding to HCVpp and HCVcc by augmenting virus entry in an SR-BI-dependent fashion (35). HDL activation of target cells accelerated virus entry by removing a 1-h lag during virus internalization. This augmentation of virus entry resulted in decreased binding of neutralizing antibodies to the CD81 binding site on E2, potentially due to limited exposure time of these epitopes. Antibodies targeted to E1E2 complex epitopes were not affected (35). This effect was mediated by the lipid transfer function of SR-BI, as inhibitors of SR-BI mediated lipid transfer fully restored the neutralizing ability of antibodies targeting the CD81 binding site. Part of the accelerated entry efficiency is potentially due to the enhancing ability of ApoC-I which has been shown to be affected by SR-BI mediated lipid transfer (86). Incorporation of ApoC-I increased the infectivity of HCV pseudoparticles after incubation with old world nonhuman primate or human sera. Antibodies against ApoC-I abrogated the enhancing activity of human serum showing that ApoC-I was indeed responsible for the enhancement of infectivity (87). In contrast to ApoE, the enhancement of infectivity by ApoC-I was dependent on HVR1 (35, 86) and its interaction with SR-BI. It remains to be determined whether enhancement of infection and escape from the neutralizing antibody response are two completely independent mechanisms or whether faster virus entry limits the exposure time to neutralizing antibodies and thus mediates escape from the neutralizing antibody response.

In addition, apolipoproteins might not only be involved in the escape from adaptive immune responses. Experimental evidence suggests that ApoE3 also mediates escape from the innate effector molecule Ficolin-2 that blocks HCV entry at an early time point during infection (88). ApoE3 indirectly blocked the interaction of Ficolin-2 and E2, even when HCVcc were preincubated with Ficolin-2, potentially due to the higher affinity of ApoE3 for the viral envelope protein (88). This underlines the important function of apolipoproteins for the evasion from the host immune response.

Taken together, apolipoproteins contribute to viral escape by two different mechanisms. Association of HCV particles with lipid components in LVPs directly inhibits neutralization by anti-envelope antibodies. In addition, interaction with apolipoproteins enhances viral entry, which limits the exposure of the virus to neutralizing antibodies. Understanding the mechanisms by which HCV usurps apolipoproteins for viral escape might offer new strategies for antiviral intervention and could pave the way toward the development of a protective vaccine.

## Impact for Antiviral Therapies and Vaccine Design

The close association of HCV with the host lipid metabolism has several important implications for HCV treatment and vaccine design. Modulation of the apolipoprotein–HCV interaction may open new opportunities for antiviral therapies and vaccines: targeting the interaction sites of apolipoproteins and viral envelope proteins could be an approach to block HCV infection and at the same time to restrict its capacity to evade from the neutralizing antibody response. In particular, viral variants isolated from patients undergoing liver transplantation are characterized by efficient viral escape (89, 90), which has been partially attributed to their incorporation of ApoE (60). Supporting this concept, Avasimibe, a clinically approved inhibitor of lipid transportation that leads to decreased ApoB and ApoE serum secretion showed broad pan-genotypic inhibition of HCV infection (91). Second, the interaction of apolipoproteins and HCV proteins provides an opportunity to identify epitopes for broadly neutralizing antibodies. Antibodies that target conserved conformational structures are less prone to mutations. However, antibodies directed against host epitopes might also open the risk for autoimmune diseases, as it has been reported for the development of autoimmune-antibodies directed against ApoA-I during HCV infection (92). This also has to be considered while choosing the correct system for vaccine production. While production in hepatic cell lines, in particular in HepG2 cells would lead to correctly lipidized LVPs that might be the best option to generate a suitable immune response, masked epitopes or the risk for autoimmune diseases, as discussed earlier, might favor different production systems such as CHO cells or yeast, as for the hepatitis B virus and human papillomavirus vaccines. It remains to be determined which production system is suitable to generate a correct immune response against HCV. Studies on chronic viral infection in the lymphocytic choriomeningitis mouse model revealed that chronic infection and the associated chronic inflammation resulted in the formation of persistent immune complexes. The complexes resulted in a dampened Fc-mediated effector activity, potentially impacting antibody-based treatment options also for HCV and other chronic viral infections (93). Furthermore, HIV vaccine trials showed that post-translational modifications, such as glycosylations that greatly depend on the production system are of utmost importance for vaccine efficacy (94–96).

Another promising approach might be the development of monoclonal antibodies targeting E1 since the shielding function of apolipoproteins was primarily directed against epitopes located on the envelope protein E2. Finally, a detailed understanding of the HCV–lipoprotein–antibody interactions may help to design immunogens inducing broadly neutralizing antibodies for protection of infection and may guide the way toward the development of a protective HCV vaccine.

## Author Contributions

All authors listed have made a substantial, direct, and intellectual contribution to the work and approved it for publication.

## Conflict of Interest Statement

The authors declare that the research was conducted in the absence of any commercial or financial relationships that could be construed as a potential conflict of interest.

## References

[1] Billerbeck E Wolfisberg R Fahnoe U Xiao JW Quirk C Luna JM Mouse models of acute and chronic hepacivirus infection. Science (2017) 357(6347):204–8. 10.1126/science.aal1962 28706073 PMC5654634

[2] WHO. Hepatitis C fact sheet. (2018). [cited 2018 04.18.]. Available from: http://www.who.int/mediacentre/factsheets/fs164/en/ (Accessed: April 18, 2018).

[3] Chung RT Baumert TF Curing chronic hepatitis C – the arc of a medical triumph. N Engl J Med (2014) 370(17):1576–8. 10.1056/NEJMp1400986 24720678

[4] Bartenschlager R Baumert TF Bukh J Houghton M Lemon SM Lindenbach BD Critical challenges and emerging opportunities in hepatitis C virus research in an era of potent antiviral therapy: considerations for scientists and funding agencies. Virus Res (2018) 248:53–62. 10.1016/j.virusres.2018.02.016 29477639

[5] Pierce BG Keck ZY Foung SK. Viral evasion and challenges of hepatitis C virus vaccine development. Curr Opin Virol (2016) 20:55–63. 10.1016/j.coviro.2016.09.004 27657659 PMC5102773

[6] Lindenbach BD. Virion assembly and release. Curr Top Microbiol Immunol (2013) 369:199–218. 10.1007/978-3-642-27340-7_8 23463202 PMC3925669

[7] Felmlee DJ Hafirassou ML Lefevre M Baumert TF Schuster C Hepatitis C virus, cholesterol and lipoproteins – impact for the viral life cycle and pathogenesis of liver disease. Viruses (2013) 5(5):1292–324. 10.3390/v5051292 23698400 PMC3712309

[8] Lavie M Dubuisson J. Interplay between hepatitis C virus and lipid metabolism during virus entry and assembly. Biochimie (2017) 141:62–9. 10.1016/j.biochi.2017.06.009 28630011

[9] Thomssen R Bonk S Propfe C Heermann KH Köchel HG Uy A Association of hepatitis C virus in human sera with beta-lipoprotein. Med Microbiol Immunol (1992) 181:293–300. 1335546 10.1007/BF00198849

[10] André P Komurian-Pradel F Deforges S Perret M Berland JL Sodoyer M Characterization of low- and very-low-density hepatitis C virus RNA-containing particles. J Virol (2002) 76:6919–28. 10.1128/JVI.76.14.6919-6928.2002 12072493 PMC136313

[11] Nielsen SU Bassendine MF Burt AD Martin C Pumeechockchai W Toms GL. Association between hepatitis C virus and very-low-density lipoprotein (VLDL)/LDL analyzed in iodixanol density gradients. J Virol (2006) 80:2418–28. 10.1128/JVI.80.5.2418-2428.2006 16474148 PMC1395398

[12] Gastaminza P Cheng G Wieland S Zhong J Liao W Chisari FV. Cellular determinants of hepatitis C virus assembly, maturation, degradation, and secretion. J Virol (2008) 82:2120–9. 10.1128/JVI.02053-07 18077707 PMC2258938

[13] Meunier J-C Russell RS Engle RE Faulk KN Purcell RH Emerson SU. Apolipoprotein c1 association with hepatitis C virus. J Virol (2008) 82:9647–56. 10.1128/JVI.00914-08 18667498 PMC2546963

[14] Catanese MT Uryu K Kopp M Edwards TJ Andrus L Rice WJ Ultrastructural analysis of hepatitis C virus particles. Proc Natl Acad Sci U S A (2013) 110:9505–10. 10.1073/pnas.1307527110 23690609 PMC3677472

[15] Piver E Boyer A Gaillard J Bull A Beaumont E Roingeard P Ultrastructural organisation of HCV from the bloodstream of infected patients revealed by electron microscopy after specific immunocapture. Gut (2017) 66:1487–95. 10.1136/gutjnl-2016-311726 27729393

[16] Gastaminza P Kapadia SB Chisari FV. Differential biophysical properties of infectious intracellular and secreted hepatitis C virus particles. J Virol (2006) 80:11074–81. 10.1128/JVI.01150-06 16956946 PMC1642172

[17] Lindenbach BD Meuleman P Ploss A Vanwolleghem T Syder AJ McKeating JA Cell culture-grown hepatitis C virus is infectious in vivo and can be recultured in vitro. Proc Natl Acad Sci U S A (2006) 103(10):3805–9. 10.1073/pnas.0511218103 16484368 PMC1533780

[18] Merz A Long G Hiet M-S Brügger B Chlanda P Andre P Biochemical and morphological properties of hepatitis C virus particles and determination of their lipidome. J Biol Chem (2011) 286:3018–32. 10.1074/jbc.M110.175018 21056986 PMC3024796

[19] Crouchet E Baumert TF Schuster C. Hepatitis C virus-apolipoprotein interactions: molecular mechanisms and clinical impact. Expert Rev Proteomics (2017) 14(7):593–606. 10.1080/14789450.2017.1344102 28625086 PMC6138823

[20] Sundaram M Yao Z. Intrahepatic role of exchangeable apolipoproteins in lipoprotein assembly and secretion. Arterioscler Thromb Vasc Biol (2012) 32:1073–8. 10.1161/ATVBAHA.111.241455 22517365

[21] Ramasamy I. Recent advances in physiological lipoprotein metabolism. Clin Chem Lab Med (2014) 52:1695–727. 10.1515/cclm-2013-0358 23940067

[22] Aizawa Y Seki N Nagano T Abe H. Chronic hepatitis C virus infection and lipoprotein metabolism. World J Gastroenterol (2015) 21:10299–313. 10.3748/wjg.v21.i36.10299 26420957 PMC4579877

[23] Jiang J Cun W Wu X Shi Q Tang H Luo G. Hepatitis C virus attachment mediated by apolipoprotein E binding to cell surface heparan sulfate. J Virol (2012) 86:7256–67. 10.1128/JVI.07222-11 22532692 PMC3416335

[24] Shi Q Jiang J Luo G. Syndecan-1 serves as the major receptor for attachment of hepatitis C virus to the surfaces of hepatocytes. J Virol (2013) 87:6866–75. 10.1128/JVI.03475-12 23576506 PMC3676102

[25] Lefevre M Felmlee DJ Parnot M Baumert TF Schuster C. Syndecan 4 is involved in mediating HCV entry through interaction with lipoviral particle-associated apolipoprotein E. PLoS One (2014) 9(4):e95550. 10.1371/journal.pone.0095550 24751902 PMC3994096

[26] Xu Y Martinez P Séron K Luo G Allain F Dubuisson J Characterization of hepatitis C virus interaction with heparan sulfate proteoglycans. J Virol (2015) 89:3846–58. 10.1128/JVI.03647-14 25609801 PMC4403428

[27] Grigorov B Reungoat E Gentil Dit Maurin A Varbanov M Blaising J Michelet M Hepatitis C virus infection propagates through interactions between Syndecan-1 and CD81 and impacts the hepatocyte glycocalyx. Cell Microbiol (2017) 19(5):e12711. 10.1111/cmi.12711 27930836

[28] Chang K-S Jiang J Cai Z Luo G. Human apolipoprotein e is required for infectivity and production of hepatitis C virus in cell culture. J Virol (2007) 81:13783–93. 10.1128/JVI.01091-07 17913825 PMC2168882

[29] Liu S McCormick KD Zhao W Zhao T Fan D Wang T. Human apolipoprotein E peptides inhibit hepatitis C virus entry by blocking virus binding. Hepatology (2012) 56:484–91. 10.1002/hep.25665 22334503 PMC3362681

[30] Owen DM Huang H Ye J Gale M. Apolipoprotein E on hepatitis C virion facilitates infection through interaction with low-density lipoprotein receptor. Virology (2009) 394:99–108. 10.1016/j.virol.2009.08.037 19751943 PMC2767442

[31] Scarselli E Ansuini H Cerino R Roccasecca RM Acali S Filocamo G The human scavenger receptor class B type I is a novel candidate receptor for the hepatitis C virus. EMBO J (2002) 21:5017–25. 10.1093/emboj/cdf529 12356718 PMC129051

[32] Zahid MN Turek M Xiao F Thi VL Guerin M Fofana I The postbinding activity of scavenger receptor class B type I mediates initiation of hepatitis C virus infection and viral dissemination. Hepatology (2013) 57(2):492–504. 10.1002/hep.26097 23081796

[33] Maillard P Huby T Andreo U Moreau M Chapman J Budkowska A. The interaction of natural hepatitis C virus with human scavenger receptor SR-BI/Cla1 is mediated by ApoB-containing lipoproteins. FASEB J (2006) 20(6):735–7. 10.1096/fj.05-4728fje 16476701

[34] Dao Thi VL Granier C Zeisel MB Guerin M Mancip J Granio O Characterization of hepatitis C virus particle subpopulations reveals multiple usage of the scavenger receptor BI for entry steps. J Biol Chem (2012) 287(37):31242–57. 10.1074/jbc.M112.365924 22767607 PMC3438956

[35] Dreux M Pietschmann T Granier C Voisset C Ricard-Blum S Mangeot PE High density lipoprotein inhibits hepatitis C virus-neutralizing antibodies by stimulating cell entry via activation of the scavenger receptor BI. J Biol Chem (2006) 281(27):18285–95. 10.1074/jbc.M602706200 16675450

[36] Thomssen R Bonk S. Virolytic action of lipoprotein lipase on hepatitis C virus in human sera. Med Microbiol Immunol (2002) 191:17–24. 10.1007/s00430-001-0106-x 12137195

[37] Shimizu Y Hishiki T Sugiyama K Ogawa K Funami K Kato A Lipoprotein lipase and hepatic triglyceride lipase reduce the infectivity of hepatitis C virus (HCV) through their catalytic activities on HCV-associated lipoproteins. Virology (2010) 407:152–9. 10.1016/j.virol.2010.08.011 20822787

[38] Sun H-Y Lin C-C Lee J-C Wang S-W Cheng P-N Wu I-C Very low-density lipoprotein/lipo-viro particles reverse lipoprotein lipase-mediated inhibition of hepatitis C virus infection via apolipoprotein C-III. Gut (2013) 62:1193–203. 10.1136/gutjnl-2011-301798 22689516

[39] Paul D Madan V Bartenschlager R. Hepatitis C virus RNA replication and assembly: living on the fat of the land. Cell Host Microbe (2014) 16:569–79. 10.1016/j.chom.2014.10.008 25525790 PMC7172941

[40] Fukuhara T Ono C Puig-Basagoiti F Matsuura Y. Roles of lipoproteins and apolipoproteins in particle formation of hepatitis C virus. Trends Microbiol (2015) 23:618–29. 10.1016/j.tim.2015.07.007 26433694

[41] Huang H Sun F Owen DM Li W Chen Y Gale M Hepatitis C virus production by human hepatocytes dependent on assembly and secretion of very low-density lipoproteins. Proc Natl Acad Sci U S A (2007) 104:5848–53. 10.1073/pnas.0700760104 17376867 PMC1829327

[42] Icard V Diaz O Scholtes C Perrin-Cocon L Ramière C Bartenschlager R Secretion of hepatitis C virus envelope glycoproteins depends on assembly of apolipoprotein B positive lipoproteins. PLoS One (2009) 4:e4233. 10.1371/journal.pone.0004233 19156195 PMC2617766

[43] Jiang J Luo G. Apolipoprotein E but not B is required for the formation of infectious hepatitis C virus particles. J Virol (2009) 83:12680–91. 10.1128/JVI.01476-09 19793818 PMC2786834

[44] Coller KE Heaton NS Berger KL Cooper JD Saunders JL Randall G. Molecular determinants and dynamics of hepatitis C virus secretion. PLoS Pathog (2012) 8:e1002466. 10.1371/journal.ppat.1002466 22241992 PMC3252379

[45] Bartenschlager R Penin F Lohmann V Andre P. Assembly of infectious hepatitis C virus particles. Trends Microbiol (2011) 19(2):95–103. 10.1016/j.tim.2010.11.005 21146993

[46] Da Costa D Turek M Felmlee DJ Girardi E Pfeffer S Long G Reconstitution of the entire hepatitis C virus life cycle in nonhepatic cells. J Virol (2012) 86(21):11919–25. 10.1128/JVI.01066-12 22896615 PMC3486316

[47] Hueging K Doepke M Vieyres G Bankwitz D Frentzen A Doerrbecker J Apolipoprotein E codetermines tissue tropism of hepatitis C virus and is crucial for viral cell-to-cell transmission by contributing to a postenvelopment step of assembly. J Virol (2014) 88(3):1433–46. 10.1128/JVI.01815-13 24173232 PMC3911621

[48] Gondar V Molina-Jimenez F Hishiki T Garcia-Buey L Koutsoudakis G Shimotohno K Apolipoprotein E, but not apolipoprotein B, is essential for efficient cell-to-cell transmission of hepatitis C virus. J Virol (2015) 89(19):9962–73. 10.1128/JVI.00577-15 26202245 PMC4577890

[49] Fukuhara T Wada M Nakamura S Ono C Shiokawa M Yamamoto S Amphipathic α-helices in apolipoproteins are crucial to the formation of infectious hepatitis C virus particles. PLoS Pathog (2014) 10:e1004534. 10.1371/journal.ppat.1004534 25502789 PMC4263759

[50] Hueging K Weller R Doepke M Vieyres G Todt D Wölk B Several human liver cell expressed apolipoproteins complement HCV virus production with varying efficacy conferring differential specific infectivity to released viruses. PLoS One (2015) 10:e0134529. 10.1371/journal.pone.0134529 26226615 PMC4520612

[51] Puig-Basagoiti F Fukuhara T Tamura T Ono C Uemura K Kawachi Y Human cathelicidin compensates for the role of apolipoproteins in hepatitis C virus infectious particle formation. J Virol (2016) 90(19):8464–77. 10.1128/JVI.00471-16 27440892 PMC5021414

[52] Fukuhara T Tamura T Ono C Shiokawa M Mori H Uemura K Host-derived apolipoproteins play comparable roles with viral secretory proteins Erns and NS1 in the infectious particle formation of Flaviviridae. PLoS Pathog (2017) 13(6):e1006475. 10.1371/journal.ppat.1006475 28644867 PMC5500379

[53] Benga WJ Krieger SE Dimitrova M Zeisel MB Parnot M Lupberger J Apolipoprotein E interacts with hepatitis C virus nonstructural protein 5A and determines assembly of infectious particles. Hepatology (2010) 51(1):43–53. 10.1002/hep.23278 20014138

[54] Cun W Jiang J Luo G. The C-terminal alpha-helix domain of apolipoprotein E is required for interaction with nonstructural protein 5A and assembly of hepatitis C virus. J Virol (2010) 84(21):11532–41. 10.1128/JVI.01021-10 20719944 PMC2953147

[55] Boyer A Dumans A Beaumont E Etienne L Roingeard P Meunier J-C. The association of hepatitis C virus glycoproteins with apolipoproteins E and B early in assembly is conserved in lipoviral particles. J Biol Chem (2014) 289:18904–13. 10.1074/jbc.M113.538256 24838241 PMC4081931

[56] Lee J-Y Acosta EG Stoeck IK Long G Hiet M-S Mueller B Apolipoprotein E likely contributes to a maturation step of infectious hepatitis C virus particles and interacts with viral envelope glycoproteins. J Virol (2014) 88:12422–37. 10.1128/JVI.01660-14 25122793 PMC4248909

[57] Li Z Li Y Bi Y Zhang H Yao Y Li Q Extracellular interactions between hepatitis C virus and secreted apolipoprotein E. J Virol (2017) 91(15):e02227-16. 10.1128/JVI.02227-16 28539442 PMC5512249

[58] Yang Z Wang X Chi X Zhao F Guo J Ma P Neglected but important role of apolipoprotein E exchange in hepatitis C virus infection. J Virol (2016) 90:9632–43. 10.1128/JVI.01353-16 27535051 PMC5068516

[59] Bankwitz D Doepke M Hueging K Weller R Bruening J Behrendt P Maturation of secreted HCV particles by incorporation of secreted ApoE protects from antibodies by enhancing infectivity. J Hepatol (2017) 67(3):480–9. 10.1016/j.jhep.2017.04.010 28438690

[60] Fauvelle C Felmlee DJ Crouchet E Lee J Heydmann L Lefèvre M Apolipoprotein E mediates evasion from hepatitis C virus neutralizing antibodies. Gastroenterology (2016) 150(1):206–17.e4. 10.1053/j.gastro.2015.09.014 26404951

[61] Nakamuta M Yada R Fujino T Yada M Higuchi N Tanaka M Changes in the expression of cholesterol metabolism-associated genes in HCV-infected liver: a novel target for therapy? Int J Mol Med (2009) 24(6):825–8. 10.3892/ijmm_00000299 19885625

[62] Fujino T Nakamuta M Yada R Aoyagi Y Yasutake K Kohjima M Expression profile of lipid metabolism-associated genes in hepatitis C virus-infected human liver. Hepatol Res (2010) 40:923–9. 10.1111/j.1872-034X.2010.00700.x 20887597

[63] Onat A Kaya A Ademoglu E. Modified risk associations of lipoproteins and apolipoproteins by chronic low-grade inflammation. Expert Rev Cardiovasc Ther (2018) 16(1):39–48. 10.1080/14779072.2018.1417839 29241386

[64] Seki N Sugita T Aida Y Itagaki M Ishiguro H Sutoh S Assessment of the features of serum apolipoprotein profiles in chronic HCV infection: difference between HCV genotypes 1b and 2. Hepatol Int (2014) 8:550–9. 10.1007/s12072-014-9572-2 26202760

[65] Kim E Li K Lieu C Tong S Kawai S Fukutomi T Expression of apolipoprotein C-IV is regulated by Ku antigen/peroxisome proliferator-activated receptor gamma complex and correlates with liver steatosis. J Hepatol (2008) 49:787–98. 10.1016/j.jhep.2008.06.029 18809223 PMC2644636

[66] Ge D Fellay J Thompson AJ Simon JS Shianna KV Urban TJ Genetic variation in IL28B predicts hepatitis C treatment-induced viral clearance. Nature (2009) 461:399–401. 10.1038/nature08309 19684573

[67] Thomas DL Thio CL Martin MP Qi Y Ge D O’Huigin C Genetic variation in IL28B and spontaneous clearance of hepatitis C virus. Nature (2009) 461:798–801. 10.1038/nature08463 19759533 PMC3172006

[68] Li JH Lao XQ Tillmann HL Rowell J Patel K Thompson A Interferon-lambda genotype and low serum low-density lipoprotein cholesterol levels in patients with chronic hepatitis C infection. Hepatology (2010) 51:1904–11. 10.1002/hep.23592 20235331 PMC2921623

[69] Harada R Kimura M Sato Y Taniguchi T Tomonari T Tanaka T APOB codon 4311 polymorphism is associated with hepatitis C virus infection through altered lipid metabolism. BMC Gastroenterol (2018) 18:24. 10.1186/s12876-018-0747-5 29382324 PMC5791310

[70] Pileri P Uematsu Y Campagnoli S Galli G Falugi F Petracca R Binding of hepatitis C virus to CD81. Science (1998) 282(5390):938–41. 10.1126/science.282.5390.938 9794763

[71] Pierce BG Keck ZY Lau P Fauvelle C Gowthaman R Baumert TF Global mapping of antibody recognition of the hepatitis C virus E2 glycoprotein: implications for vaccine design. Proc Natl Acad Sci U S A (2016) 113(45):e6946–54. 10.1073/pnas.1614942113 27791171 PMC5111724

[72] Fuerst TR Pierce BG Keck ZY Foung SKH Designing a B cell-based vaccine against a highly variable hepatitis C virus. Front Microbiol (2017) 8:2692 10.3389/fmicb.2017.02692 29379486 PMC5775222

[73] Giang E Dorner M Prentoe JC Dreux M Evans MJ Bukh J Human broadly neutralizing antibodies to the envelope glycoprotein complex of hepatitis C virus. Proc Natl Acad Sci U S A (2012) 109(16):6205–10. 10.1073/pnas.1114927109 22492964 PMC3341081

[74] Guan M Wang W Liu X Tong Y Liu Y Ren H Three different functional microdomains in the hepatitis C virus hypervariable region 1 (HVR1) mediate entry and immune evasion. J Biol Chem (2012) 287(42):35631–45. 10.1074/jbc.M112.382341 22927442 PMC3471721

[75] Barth H Schafer C Adah MI Zhang F Linhardt RJ Toyoda H Cellular binding of hepatitis C virus envelope glycoprotein E2 requires cell surface heparan sulfate. J Biol Chem (2003) 278(42):41003–12. 10.1074/jbc.M302267200 12867431

[76] Barth H Schnober EK Zhang F Linhardt RJ Depla E Boson B Viral and cellular determinants of the hepatitis C virus envelope-heparan sulfate interaction. J Virol (2006) 80(21):10579–90. 10.1128/JVI.00941-06 16928753 PMC1641783

[77] Sabo MC Luca VC Prentoe J Hopcraft SE Blight KJ Yi M Neutralizing monoclonal antibodies against hepatitis C virus E2 protein bind discontinuous epitopes and inhibit infection at a postattachment step. J Virol (2011) 85(14):7005–19. 10.1128/JVI.00586-11 21543495 PMC3126585

[78] Mankowski MC Kinchen VJ Wasilewski LN Flyak AI Ray SC Crowe JE Jr Synergistic anti-HCV broadly neutralizing human monoclonal antibodies with independent mechanisms. Proc Natl Acad Sci U S A (2018) 115(1):E82–91. 10.1073/pnas.1718441115 29255018 PMC5776832

[79] Di Lorenzo C Angus AG Patel AH. Hepatitis C virus evasion mechanisms from neutralizing antibodies. Viruses (2011) 3(11):2280–300. 10.3390/v3112280 22163345 PMC3230852

[80] Bradley D McCaustland K Krawczynski K Spelbring J Humphrey C Cook EH. Hepatitis C virus: buoyant density of the factor VIII-derived isolate in sucrose. J Med Virol (1991) 34(3):206–8. 10.1002/jmv.1890340315 1655970

[81] Molina S Castet V Fournier-Wirth C Pichard-Garcia L Avner R Harats D The low-density lipoprotein receptor plays a role in the infection of primary human hepatocytes by hepatitis C virus. J Hepatol (2007) 46(3):411–9. 10.1016/j.jhep.2006.09.024 17156886

[82] Thomssen R Bonk S Thiele A. Density heterogeneities of hepatitis C virus in human sera due to the binding of beta-lipoproteins and immunoglobulins. Med Microbiol Immunol (1993) 182(6):329–34. 10.1007/BF00191948 8121333

[83] Tao W Xu C Ding Q Li R Xiang Y Chung J A single point mutation in E2 enhances hepatitis C virus infectivity and alters lipoprotein association of viral particles. Virology (2009) 395(1):67–76. 10.1016/j.virol.2009.09.006 19793603

[84] Grove J Nielsen S Zhong J Bassendine MF Drummer HE Balfe P Identification of a residue in hepatitis C virus E2 glycoprotein that determines scavenger receptor BI and CD81 receptor dependency and sensitivity to neutralizing antibodies. J Virol (2008) 82(24):12020–9. 10.1128/JVI.01569-08 18829747 PMC2593310

[85] Weller R Hueging K Brown RJP Todt D Joecks S Vondran FWR Hepatitis C virus strain-dependent usage of apolipoprotein E modulates assembly efficiency and specific infectivity of secreted virions. J Virol (2017) 91(18):e00422-17. 10.1128/JVI.00422-17 28659481 PMC5571276

[86] Dreux M Boson B Ricard-Blum S Molle J Lavillette D Bartosch B The exchangeable apolipoprotein ApoC-I promotes membrane fusion of hepatitis C virus. J Biol Chem (2007) 282(44):32357–69. 10.1074/jbc.M705358200 17761674

[87] Meunier JC Engle RE Faulk K Zhao M Bartosch B Alter H Evidence for cross-genotype neutralization of hepatitis C virus pseudo-particles and enhancement of infectivity by apolipoprotein C1. Proc Natl Acad Sci U S A (2005) 102(12):4560–5. 10.1073/pnas.0501275102 15767578 PMC555507

[88] Zhao Y Ren Y Zhang X Zhao P Tao W Zhong J Ficolin-2 inhibits hepatitis C virus infection, whereas apolipoprotein E3 mediates viral immune escape. J Immunol (2014) 193(2):783–96. 10.4049/jimmunol.1302563 24928988

[89] Fafi-Kremer S Fofana I Soulier E Carolla P Meuleman P Leroux-Roels G Viral entry and escape from antibody-mediated neutralization influence hepatitis C virus reinfection in liver transplantation. J Exp Med (2010) 207(9):2019–31. 10.1084/jem.20090766 20713596 PMC2931157

[90] Fofana I Fafi-Kremer S Carolla P Fauvelle C Zahid MN Turek M Mutations that alter use of hepatitis C virus cell entry factors mediate escape from neutralizing antibodies. Gastroenterology (2012) 143(1):223–33.e9. 10.1053/j.gastro.2012.04.006 22503792 PMC5295797

[91] Hu L Li J Cai H Yao W Xiao J Li YP Avasimibe: a novel hepatitis C virus inhibitor that targets the assembly of infectious viral particles. Antiviral Res (2017) 148:5–14. 10.1016/j.antiviral.2017.10.016 29074218

[92] Bridge SH Pagano S Jones M Foster GR Neely D Vuilleumier N Autoantibody to apolipoprotein A-1 in hepatitis C virus infection: a role in atherosclerosis? Hepatol Int (2018) 12(1):17–25. 10.1007/s12072-018-9842-5 29423541 PMC5814532

[93] Gunn BM Alter G. Modulating antibody functionality in infectious disease and vaccination. Trends Mol Med (2016) 22(11):969–82. 10.1016/j.molmed.2016.09.002 27756530 PMC8767654

[94] Demberg T Robert-Guroff M. B-cells and the use of non-human primates for evaluation of HIV vaccine candidates. Curr HIV Res (2015) 13(6):462–78. 10.2174/1570162X13666150724095339 26206458

[95] Wren LH Stratov I Kent SJ Parsons MS. Obstacles to ideal anti-HIV antibody-dependent cellular cytotoxicity responses. Vaccine (2013) 31(47):5506–17. 10.1016/j.vaccine.2013.08.035 23981432

[96] Mahan AE Jennewein MF Suscovich T Dionne K Tedesco J Chung AW Antigen-specific antibody glycosylation is regulated via vaccination. PLoS Pathog (2016) 12(3):e1005456. 10.1371/journal.ppat.1005456 26982805 PMC4794126

